# The Role of Adsorbed and Subsurface Carbon Species for the Selective Alkyne Hydrogenation Over a Pd-Black Catalyst: An *Operando* Study of Bulk and Surface

**DOI:** 10.1007/s11244-018-1071-6

**Published:** 2018-10-24

**Authors:** J. J. Velasco-Vélez, D. Teschner, F. Girgsdies, M. Hävecker, V. Streibel, M. G. Willinger, J. Cao, M. Lamoth, E. Frei, R. Wang, A. Centeno, A. Zurutuza, S. Hofmann, R. Schlögl, A. Knop-Gericke

**Affiliations:** 1 Department of Heterogeneous Reactions, Max Planck Institute for Chemical Energy Conversion, 45470 Mülheim an der Ruhr, Germany; 2 Department of Inorganic Chemistry, Fritz-Haber-Institut der Max-Planck-Gesellschaft, 14195 Berlin, Germany; 3 Department of Engineering, University of Cambridge, Cambridge, CB3 0FA UK; 4 Graphenea, 20018 San Sebastian, Spain

**Keywords:** Pd catalyst, Selective alkynes hydrogenation, Subsurface carbon, Operando XRD, Atmospheric XPS, Graphene membrane

## Abstract

The selective hydrogenation of propyne over a Pd-black model catalyst was investigated under *operando* conditions at 1 bar making use of advanced X-ray diffraction (bulk sensitive) and photo-electron spectroscopy (surface sensitive) techniques. It was found that the population of subsurface species controls the selective catalytic semi-hydrogenation of propyne to propylene due to the formation of surface and near-surface PdC_x_ that inhibits the participation of more reactive bulk hydrogen in the hydrogenation reaction. However, increasing the partial pressure of hydrogen reduces the population of PdC_x_ with the concomitant formation of a β-PdH_x_ phase up to the surface, which is accompanied by a lattice expansion, allowing the participation of more active bulk hydrogen which is responsible for the unselective total alkyne hydrogenation. Therefore, controlling the surface and subsurface catalyst chemistry is crucial to control the selective alkyne semi-hydrogenation.

**Electronic supplementary material:**

The online version of this article (10.1007/s11244-018-1071-6) contains supplementary material, which is available to authorized users.

## Introduction

Catalytic hydrogenation of hydrocarbons is an important family of processes in chemical industry [1]. The synthesis of alkenes and partial hydrogenation of multifunctional unsaturated hydrocarbons is of fundamental importance, both from and industrial and academic point of view [2]. These reaction routes are essential for example in polymerization processes where the complete elimination of alkynes and diolefins from the alkene feedstocks is the aim. Consequently, the selective elimination of propyne from a propylene rich stream is important to avoid the poisoning of the polymerization catalysts [3]. While many metals have been found to be able to catalyze the full hydrogenation of alkynes, Pd can perform their partial hydrogenation [4]. In this direction, Pd-based catalysts can effectively hydrogenate alkynes near room temperature. Nevertheless, other products such as alkanes and/or higher hydrocarbons, which are referred to as green oil or foulant, may be produced during the reaction [5] deactivating the catalyst over time. Even though alkyne hydrogenation is of utmost importance in the polymer industry, it is not yet fully understood due to two main difficulties: (1) the existence of a “pressure gap” between model experiments and real reaction conditions [6], (2) the reacting species in the hydrogenation reactions should be moderately adsorbed and present only during reaction conditions as opposed to strongly adsorbed species which are mostly spectators [7]. In particular the hydrogenation of alkenes does not depend on the catalyst structure with the weakly bonded subsurface hydrogen as key for the hydrogenation reaction occur [8, 9]. It has been found that in small particles the accessibility of the subsurface hydrogen atoms is enhanced as results of their nanoscale dimensions yielding unselective full hydrogenation process [10, 11]. Nevertheless, the question if these results can be extended to high-pressure conditions still open [12, 13]. It has been proposed in literature that the selectivity and subsurface chemistry are strongly cross-linked, as either carbon or hydrogen can occupy subsurface sites, and thus govern the selectivity in alkyne hydrogenation [14, 15]. Furthermore, it was reported that the bulk H atoms are largely reactive species in hydrogenation reactions, while H atoms bound to the surface are less reactive pointing out the importance of bulk/subsurface species in heterogeneous catalysis [16], where the presence of carbonaceous species is determinant in the catalytic performance [17]. In this way, it was found that the carbon deposition strongly influences the hydrogen depth profile distribution promoting the diffusion of hydrogen into the palladium bulk [18].Thereby, the combination of in situ spectroscopy and DFT calculations has shown that the existence of sizable barriers for hydrogen emerging from the bulk through PdC_x_ to the surface [19] is the origin for the alkynes’ selective hydrogenation. The investigations indicated that the existence of PdC_x_ at the surface strongly reduces the chemisorption energy of hydrogen and hence its coverage. On the other hand, during the unselective hydrogenation the subsurface and surface are dominated by high hydrogen population.

Here we combine a bulk sensitive technique, namely X-ray diffraction (XRD), with surface sensitive X-ray photoelectron spectroscopy (XPS) at elevated pressure (1 bar) to close the “pressure gap” and reveal the role of the surface, subsurface and bulk species in the selective hydrogenation of alkynes. We use propyne over a Pd-black catalyst as a model hydrogenation system. We find that PdC_x_ species are the key factor in the selective semi-hydrogenation as they inhibit the participation of subsurface hydrogen in the reaction. As a consequence, the equilibrium between surface, subsurface and bulk H is disturbed, giving rise to a reduced surface H concentration and, consequently, only partial alkyne hydrogenation. These results are in good agreement with previous investigations performed in our group at few mbar using the so-called ambient pressure XPS (AP-XPS) [14, 15]. Furthermore, low pressure model data (few mbar) reveals the same trends than high pressure experiments indicating that in this specific case there is no pressure gap between low and high pressure conditions. The absence of pressure gap is due to the storage function of Pd on one side and the extremely high sticky coefficient of the organic feed on the other hand building a sufficient carbon monolayer at low pressure. This carbon monolayer protects the metal and seals it from the reactive atmosphere. The high sticking is a variant of the concept of the “virtual pressure” introduced by G. Ertl indicating that under such circumstances the effective pressure is much smaller than the hydrostatic pressure [20].

## Experimental

### Paladium Black Catalyst

The Pd-black catalyst with a surface area of 40 m^2^/g (as the BET analysis shows in the SI) and 99.95% purity (trace metal basis) was sourced from Sigma-Aldrich. This catalyst was characterized using different techniques which are provided in the supplementary information (SI).

### ESEM/TEM Catalyst Characterization Measurements

Characterization of the Pd-black catalyst was conducted by both scanning electron microscopy (SEM) and transmission electron microscopy (TEM) techniques. SEM characterization was performed with a Hitachi S4800. High-resolution imaging was accomplished using a FEI TITAN 80–300 in TEM mode. In situ ESEM measurements were performed with a FEI Quanta 200 FEG electron microscopy at 1 mbar total partial pressure.

### Operando XRD

X-ray diffraction measurements were performed in situ at 1 bar and 25 °C with a STOE Bragg–Brentano Theta/Theta diffractometer (Cu Kα_1,2_ radiation, secondary graphite monochromator, scintillation detector) equipped with an Anton Paar XRK 900 reactor chamber. Precise gas supply was achieved with Bronkhorst mass flow controllers, using He as balance gas at a total flow of 100 ml/min. The outlet line is connected to a fast gas-chomatograph (VARIAN µ-GC CP4900 equipped with four independent detection channels) allowing the *on line* identification and quantification of the chemicals products evolving from the alkyne hydrogenation.

### Operando XPS

**Fig. 1:**
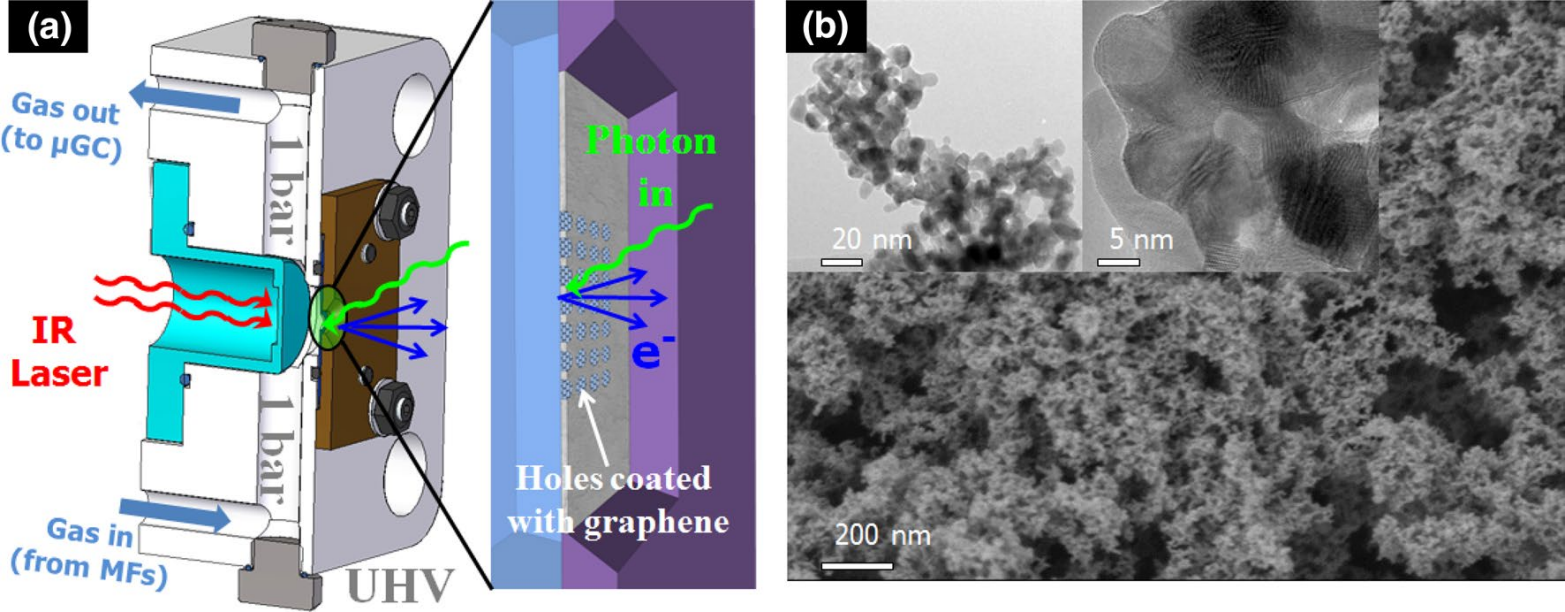
**a** Cross-sectional view of the in situ XPS gas cell including the detection scheme through a holey array microstructure coated with free standing graphene which works as an impermeable membrane for gases, that it is at the same time “transparent” for the photoelectrons. **b** SEM image of the sponge-like Pd-black catalyst used as model catalyst including the TEM images, which are included in the insets

The *operando* photoelectron spectroscopy experiments were performed in the NAP-XPS end-station of the ISISS [21] beamline (BESSY II, Berlin). In presence of gases the strong inelastic scattering of the photoelectrons with the gas molecules prevents the effective collection of photoelectrons in the analyzer. To circumvent inelastic scattering events, a photoelectron transparent membrane (graphene) was used as catalyst support allowing the separation of the reaction volume (at 1 bar) from the photoelectron analyzer (ultra-high vacuum). The chemical-vapour deposited (CVD) graphene was grown on Cu foil and transferred to the Si_3_N_4_ grids (Ted Pella Inc, Redding CA), as described elsewhere [22]. Solid Pd-black powder was used as model catalyst for the hydrogenation experiments. The powder was drop casted on the graphene membrane from Pd nanoparticles suspended in acetone on the graphene membrane on the side exposed to feed gases. Taking advantage of this concept, a reaction gas cell was developed allowing an effective control of the reaction feed gases in a flow-through scheme achieving operation pressures of up to 1 bar as shown in Fig. 1a, where the key part of the system is a holey array structure coated with free standing graphene, which solves stability problems. This membrane is used as catalyst support and at the same time it is transparent for the photoelectrons and leak tight for the gases. More details of this setup are provided elsewhere [23]. The feed gases (lab grade purity) were accurately dosed by calibrated Bronkhorst mass flow controllers, where helium was used as inert balance gas.

### Thermal Desorption Spectroscopy (TDS)

Thermal desorption spectroscopy (TDS) was applied for the temperature programmed desorption of various gases. Therefore, a self-constructed setup which enables the testing of powder samples was used. The setup is equipped with mass flow controllers, an IR-light furnace (Behr IRF 10) and a mass spectrometer (Pfeiffer Vacuum QME 200). The powder sample is placed on a small quartz-glass boat which is placed in a quartz tube (inner diameter of 14 mm, outer diameter of 20 mm, length of 450 mm) located inside of furnace and connected to the system using Ultra Torr vacuum fittings. The mass loaded was 61.6 mg. The sample is pre-treated at 1 bar at 30 °C for 3 h in 5% H_2_ in Ar with a flow of 100 ml min^−1^. The gases are detected using the mass spectrometer leak valve. Afterwards the system is stepwise brought to 9 × 10^−7^–2 × 10^−6^ mbar and directly connected to the mass spectrometer. The desorption experiment is conducted at a heating rate of 25 °C min^−1^ up to 695 °C. All masses and the temperature are monitored online.

## Results and Discussion

### Sample Characterization

Pd-black catalyst powder from Sigma-Aldrich with a surface area 40 m^2^/g, 99.95% purity (trace metal basis) was used for the in situ XRD and XPS experiments. Figure 1b shows a SEM image of the Pd-black catalyst indicating a clearly sponge-like structure with macro-/mesoporous structure frameworks and continuous closely packed aggregates of nano-crystallites as shown in the TEM, Fig. 1b insets. This structure and arrangement allows for a good diffusion of feed gas molecules and the high surface area enables high alkyne conversion facilitating the products detection. TEM measurements indicate that the primary particle size is in the range of 5–15 nm (mean size ~ 9 nm) and that the sponge-like structure comes from the aggregation of primary crystallites. In situ ESEM measurements (at 1 mbar partial pressure) of the hydrogenation of propyne onto a Pd black catalyst are shown in Fig. S4 revealing no detectable changes in the morphology but a modification in the image contrast ascribed to a variation in the work function. Thus, accurate atomistic description of the catalyst structure under reaction conditions required additional characterization techniques sensitive to small variations.

### Operando XRD Characterization

**Fig. 2:**
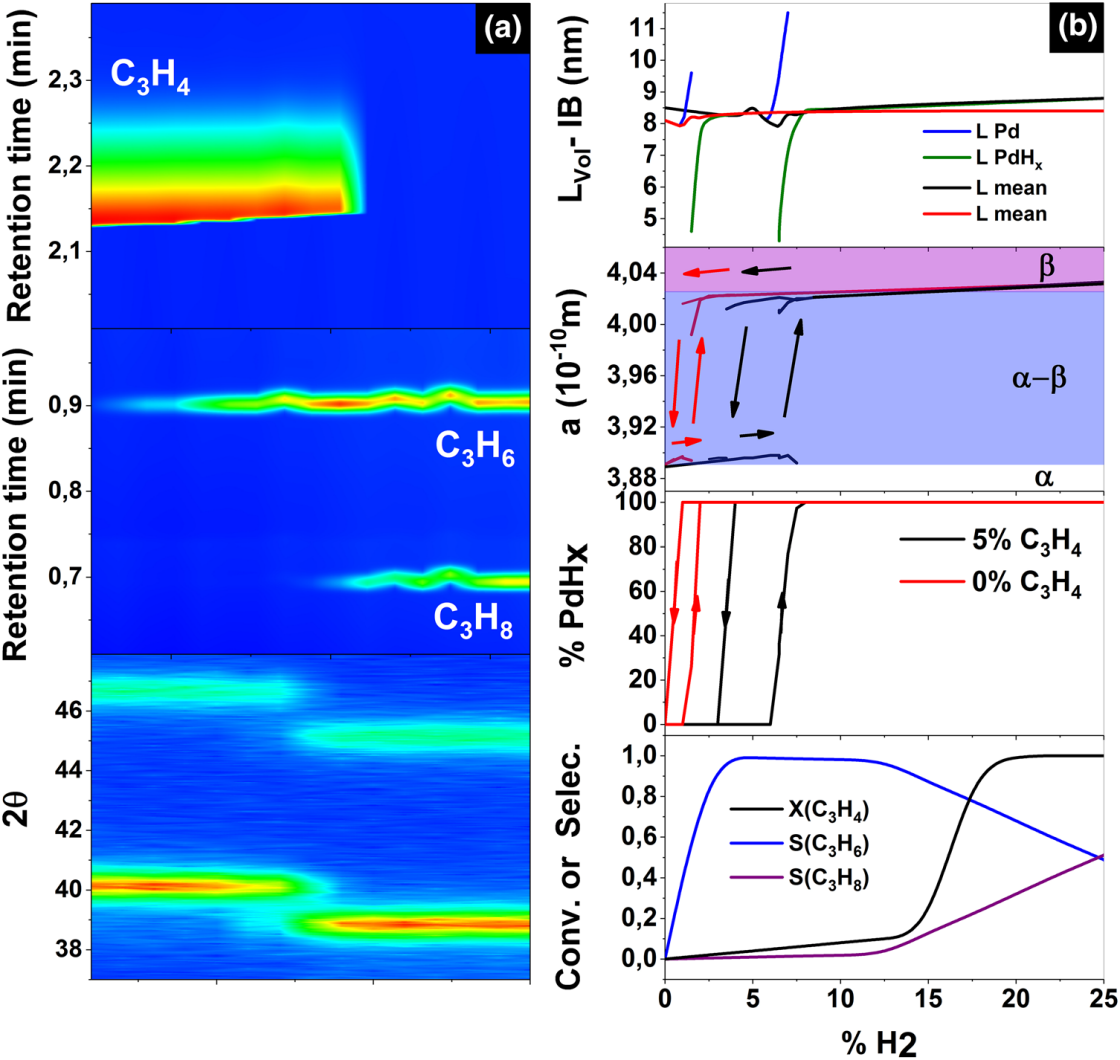
**a** Operando XRD measurements including the product of the reaction detected by GC. **b** Conversion (X) and selectivity (S) measurements and the XRD analysis of the fraction of PdH_x_, lattice constant and grain size. The XRD measurements were compared with (5% C_3_H_4_, black) and without (0% C_3_H_4_, red) versus the H_2_ concentration

The XRD measurements revealed that the formation of carbides and hydride is accompanied by the expansion of the Pd lattice [24–26]. Accordingly, hydrogen is incorporated in the face centered cubic (fcc) lattice of Pd (PdH_x_) as atomic hydrogen in the energetically favorable octahedral interstitial voids [27]. Otherwise, the formation of carburized Pd where carbon occupies the octahedral sites of the fcc lattice has been reported [28], with the near surface enrichment of carbon atoms yielding diffusion barriers for other molecules to reach the Pd bulk [25]. Here we investigate the evolution in the lattice constant of the Pd-black catalyst under reaction conditions (at room temperature, 25 °C) making use of the in situ XRD setup described in the Sect. 2 yielding valuable information in the structural changes under different reaction conditions (the operando XRD measurements including the product analysis is shown in Fig. 2a). First, two XRD patterns in 25% H_2_/75% He and pure He were recorded as references to characterize the palladium hydride and metal state, respectively. The measurement in hydrogen was conducted first to ensure that potentially present PdO_x_ traces were reduced before measuring the metal pattern. Between these two measurements, the crystallite size remained constant (within the estimated standard deviation, e.s.d.). In contrast, the lattice constant changes from 3.8872 Å (Pd metal, which is in excellent agreement with the literature value of 3.8907 Å) [29] to 4.0332 Å for PdH_x_ [30], indicating the formation of the hydrogen rich β hydride phase (0.58 < *x* < 1) under these conditions [31]. Subsequently, the sample was exposed to two feed atmospheres, containing 0% and 5% of propyne, versus a cycle of hydrogen exposition (these measurements are shown in Fig. S5). A detailed analysis of the XRD traces together with the conversion (C) and selectivity (S) measurements is shown in Fig. 2b yielding valuable information related to the percentage of PdH_x_, lattice constant (a) and crystallite size (L) variation under reaction conditions. In all cases, the crystallite size was calculated from the peak broadening. First, the Pd black catalyst performance was investigated with 0% C_3_H_4_ versus H_2_ percentage (red line) where the cycled H_2_ exposition indicates the existence of a phase transition hysteresis. Thus, increasing the concentration of hydrogen to 2% yields the formation of 100% PdH_x_ with a lattice constant equal to 4.019 Å ascribed to the formation of a α-β phase. Increasing the concentration of H_2_ to 5% yields the formation of a β phase. Reducing the concentration of H_2_ induces a change from PdH_x_ to Pd^0^ at a concentration of 0.5% H_2_ indicating that the phase transition occurs at lower hydrogen concentration due to an enhanced diffusivity of H_2_ in the bulk accompanied by an increase in the lattice constant. Thus, the strong α-phase H stabilization in the Pd nanocrystals reflects a volume property, which should account for boundary effects on their elastic properties [10]. Interestingly, even the main crystallite size remains constant (L_main_), in the overall hydrogenation process as shown in the Fig. 2b top graph, the size of the Pd^0^ crystallites (L Pd) is bigger than the size of the PdH_x_ crystallites (L PdH_x_) in average. This found indicates clearly that for the smallest crystallites the accessibility of the H to the bulk is enhanced as consequence of their small dimension yielding the full PdH_x_ at lower partial pressures of H.

The observed hysteresis in the phase indicates that the Pd bulk hydrogenation is controlled by the hydrogen diffusion in the bulk more likely than the hydrogen concentration. Based on Fig. 2b a decrease of the selectivity is observed at high H_2_:C_3_H_4_ ratios (i.e., also propane is formed). This conversion is ascribed to the existence of the α or mixed α-β phase according to the XRD measurements. For 15% and higher H_2_ concentrations, the lattice parameters shown in Fig. 2b fall into the known range of the β-PdH_x_ (*a*_min_ = 4.025 Å with x = 0.58) yielding a full conversion of propyne X(C_3_H_4_) accompanied by an unselective total hydrogenation of propyne to propane S(C_3_H_8_). Thus, at high C_3_H_4_:H_2_ ratios the selectivity changes and the catalyst produces also propane. Note, the lattice parameter value observed for H_2_ concentration lower than 15% lies below this limit β-PdH_x_ even when considering an error bar of three times the e.s.d. At the same time, it is well above the limit for the α-PdH_x_ phase (*a*_max_ = 3.895 Å with x = 0.02). These two solids display β-PdH_x_ (0.58 < *x* < 1) and α-phase (0 < *x* < 0.03) [31], where the co-existence is observed in the region from x = 0.03 to x = 0.58 [32]. Thus, the lattice parameters extracted from the peak positions and the separation of metal and hydride is clearly indicating the β-phase for all gas mixtures with a H_2_ concentration higher than 15%, as shown in Fig. 2b. The observed lattice parameter of the lowest C_3_H_4_:H_2_ feed ratio suggest a ɑ-β transition [33]. Thus, one could speculate that the presence of hydrocarbons in the feed may alter the lattice parameter by forming something like a mixed carbide/hydride phase. Whether there is carbide formation or simply a slightly different hydride composition, it is difficult to assess based on XRD measurement [34]. Nevertheless, XRD measurements corroborate that the distribution of H in the bulk depends on the partial pressure of hydrogen indicating that not only the subsurface contributes to the overall critical phase changes. It has been probed that the amount of H species in the sub-surface volume is lowered whereas the amount of H in the bulk are the same than in the case of carbon covered particles [18]. However, XRD measurements proved that only under higher partial pressures of hydrogen there is a phase transition in good agreement with changes in the unselectively to alkane hydrogenation depending also in other parameter as cristallyte size.

### Operando XPS Characterization

**Fig. 3:**
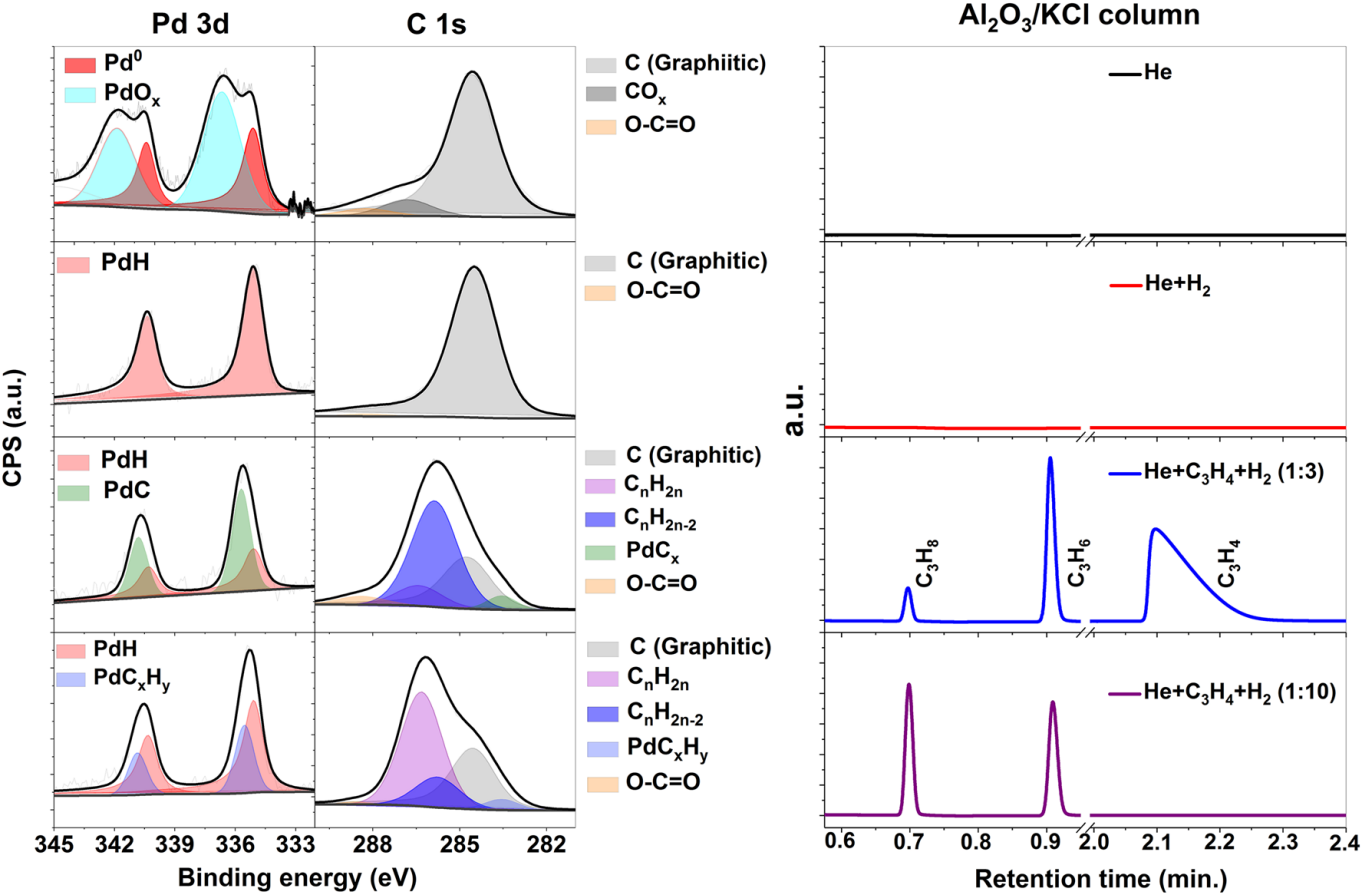
XPS and gas chromatography measurements under various conditions collected at 600 eV kinetic energy: In presence of He (black), H_2_ (red), 1:3 (C_3_H_4_:H_2_, blue) and bottom 1:10 (C_3_H_4_:H_2_, purple) in He balance 71.4%

**Fig. 4:**
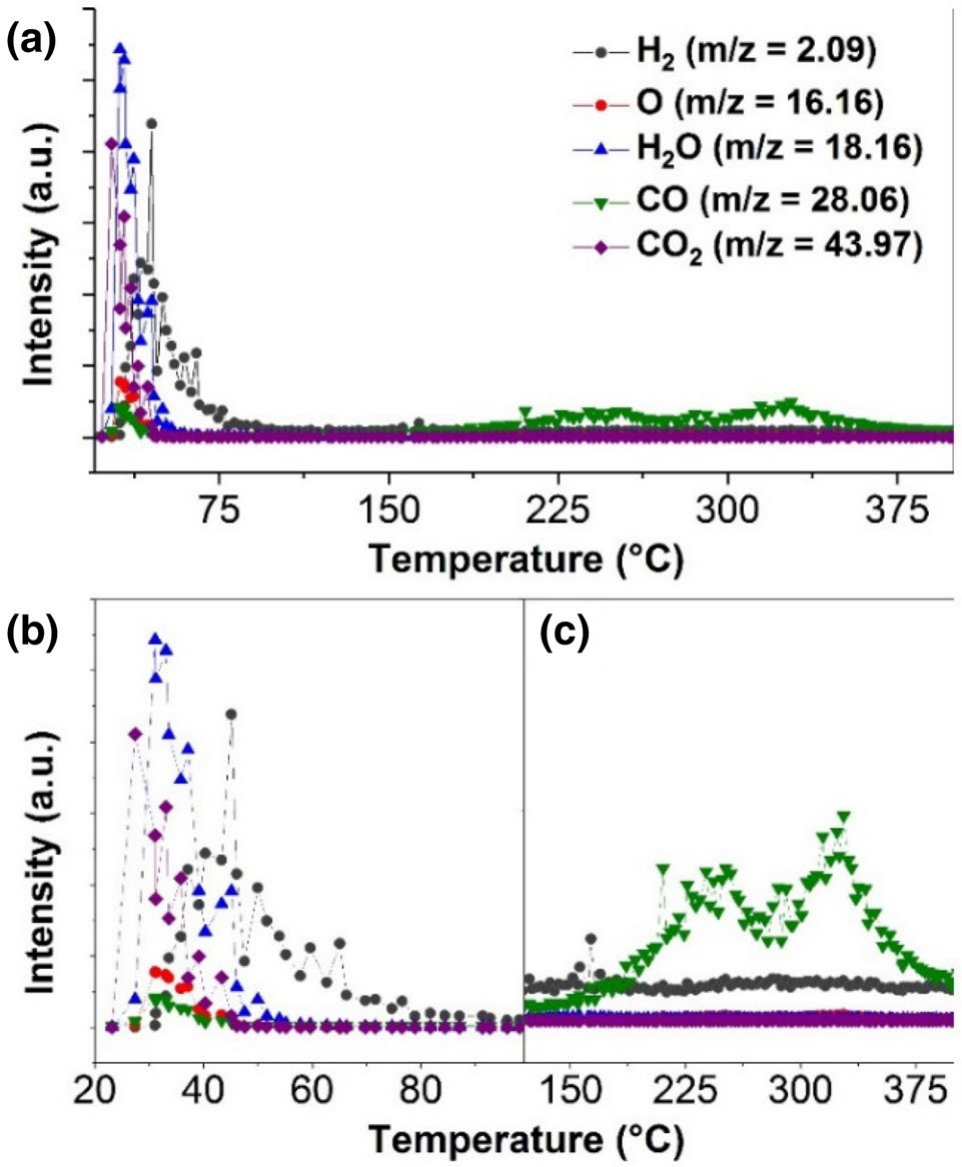
Thermal desorption spectroscopy characterization of the Pd black catalyst

The nature of the active Pd surface sites under partial and total hydrogenation at 1 bar was investigated by means of in situ XPS by recording and assessing the electronic structure information provided by the Pd 3d [35] and C 1s [36] core levels using the cell described in the Sect. 2. Figure 3 shows the XP spectra for four different gaseous environments as well as the *on-line* gas chromatography analysis of the effluent gases. As received sample, in presence of 100% He the Pd 3d shows two peaks ascribed to the Pd^0^ (335.0 eV) and PdO_x_ (336.6 eV) species. The C 1s spectra reveals the existence of a main peak ascribed to the graphitic carbon (284.6 eV) and two additional peaks ascribed to different carbonaceous species as CO (286.7 eV), and O–C=O (288.4 eV) as the TDS measurement shown. The O–C=O contribution is due to residues of PMMA from the graphene transfer process or contamination in the sample. After that, flowing a mixture of H_2_/He with a ratio of 29.6%/71.4% at room temperature the Pd 3d shows a peak at 335.0 eV (red) ascribed to the formation of PdH_x_ species [37]. Under these conditions, the C 1s shows one main peak at 284.6 eV (gray) associated with the graphitic carbon species present on the free standing graphene support and carbon on the Si_3_N_4_ wafer [38]. The peak at 288.4 eV is related to inert carbonyl groups presents in the back side of the membrane in the vacuum side and on the catalyst as the thermal desorption spectroscopy (TDS) shows in the Fig. 4. This measurements indicates the existence of physisorbed CO_2_ and H_2_O which is desorbed at around ~ 30 °C. After that the hydrogen incorporated in the Pd lattice is desorbed at around ~ 50 °C. At higher temperature (~ 200 °C) CO evolved indicating the existence of carbonyl like groups (O–C=O). Note that the XPS peak at 286.7 eV related to CO vanished in presence of H_2_ in good agreement with the TDS measurements. This measurements underlines the multi-method approach going beyond a “simple” identification of the adsorbate covering the catalyst. After that, a stream of 30 ml/min He, 9 ml/min H_2_ and 3 ml/min C_3_H_4_ (71.4%/21.4%/7.2%) was flowed yielding a relative concentration of 1:3 (C_3_H_4_:H_2_). Note, this feed provided reaction conditions still in the selective hydrogenation regime (α-β phase Fig. 2b). Accordingly, the gas chromatograph (column Al_2_O_3_/KCl; sensitive to hydrocarbons) shows the formation of C_3_H_6_ and C_3_H_8_ with higher selectivity to C_3_H_6_. Under selective hydrogenation condition a new peak in the Pd 3d spectrum at around 335.6 eV has formed, which is ascribed with the formation of PdC_x_ species on the catalyst surface. This peak is strongly related to an equivalent peak in the C 1s core level at around 283.6 eV (green peak) also associated with the formation of PdC_x_ species [14], which is characteristic for the carbon-modified Pd surface phase. In addition, there are two other peaks in the C 1s spectrum at 285.8 eV (associated to chemisorbed alkyne groups, blue peak) in a di-σ-bonded geometry [11] and 286.4 eV (attributed to chemisorbed alkene groups, purple peak) in a π-bonded geometry [12]. The chemical shift to higher binding energies in the C 1s spectra is due to a higher degree of hydrogenation in good agreement with previous results [39, 40]. Thus under a 1:3 (C_3_H_4_:H_2_) feed the dominantly species are chemisorbed C_n_H_2n−2_ groups over the PdC_x_ surface. Upon increasing the concentration of hydrogen to 1:10 (C_3_H_4_:H_2_) by flowing a mixture of 30 ml/min He, 9 ml/min H_2_ and 0.9 ml/min C_3_H_4_ (71.4%/21.4%/7.2%), the unselective propyne hydrogenation is the dominant reaction as indicated by the GC analysis (µGC column Al_2_O_3_/KCl). The change in the selectivity is accompanied by the loss of the Pd 3d peak intensity associated to the PdC_x_ near-surface phase. The peak at lower intensity at nearly the same binding energy position (marked as PdC_x_H_y_) corresponds to C_x_H_y_ adsorbate inducing core level shift [36]. Furthermore, the PdH_x_ species were enhanced on the surface as shown in Pd 3d by the red peak. The accumulation of carbonaceous deposits affects the activity and the selectivity [18]. The hydrogenation process arises from the fact that under reaction conditions it is accompanied by a decomposition of some of the reactants and concurrent deposition of partly dehydrogenated carbonaceous species on the surface [41]. The carbonaceous species resulting from the early decomposition of the reactants modify the adsorption properties of the surface and critically control the selectivity due to the inclusion of weakly or strongly bound adsorption sites yielding different reactivity toward the hydrogenation. Thus, π-bonded species are not in direct contact with the palladium surface and the alkyne desorbs intact and the hydrogenation proceeds only via di-σ-bonded alkene [12]. As a consequence PdC_x_ transforms to PdH_x_ with adsorbed hydrocarbon on the surface leading to unselective hydrogenation [42]. The presence of PdC_x_ weakens the bonding of hydrogen to the surface reducing the surface concentration being one of the major effects on the selectively hydrogenation of alkynes onto Pd black catalysts. Furthermore, the chemisorbed species associated with C_n_H_2n_ (purple) versus the C_n_H_2n−2_ (blue) are enhanced, indicating that these species on the Pd surface as well as the actual state of the surface (PdC_x_ versus PdH_x_) plays a key role in the overall reaction [43]. Note that the peak at 288.4 eV is associated to the presence of non-active carbonyl groups in the back side of the membrane and on the Pd sample.

### Discussion

**Fig. 5:**
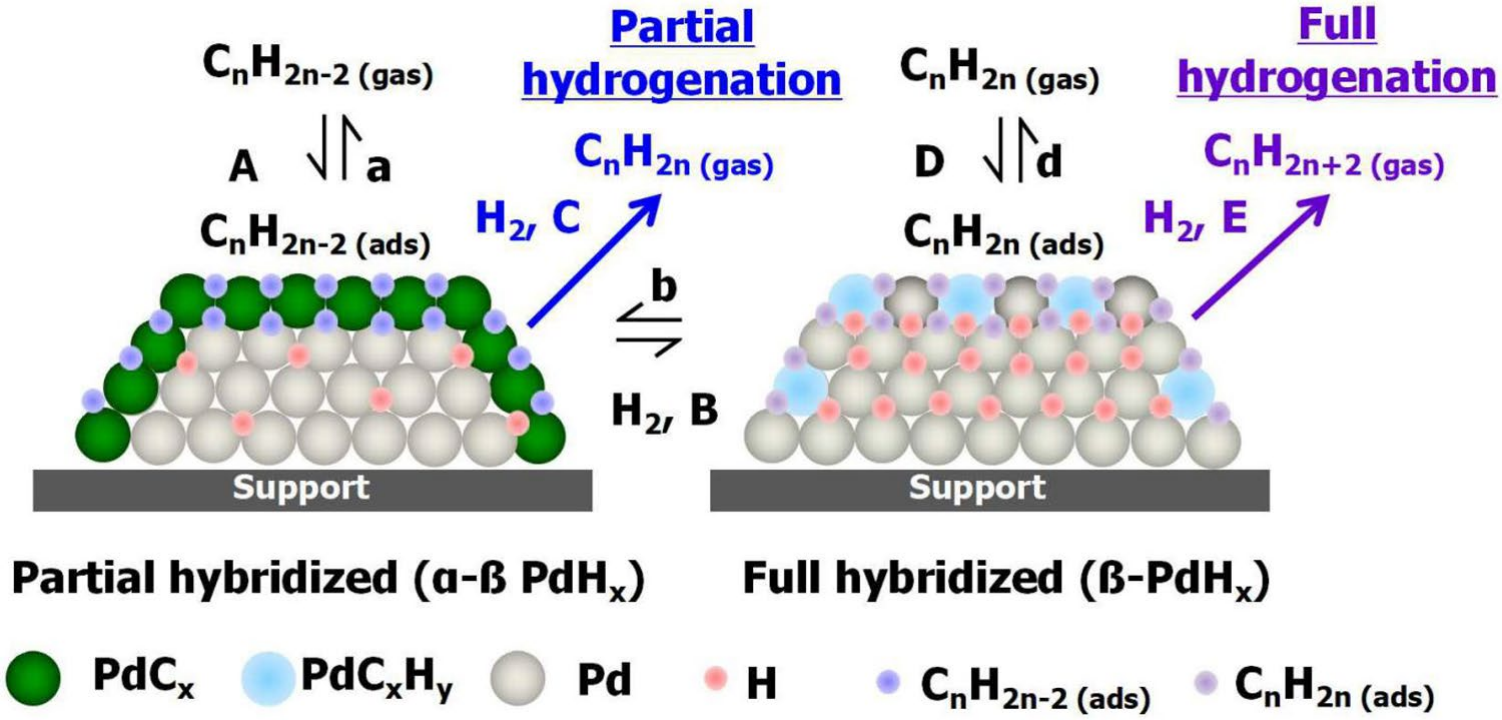
Proposed extended “rake” reaction mechanism accounting the formation of surface/subsurface PdH_x_/PdC_x_ and bulk β-PdH_x_ species as well as chemisorbed C_n_H_2n−2_/C_n_H_2n_ intermediates

According to the above described experiments, the existence of PdC_x_ species, which are formed by alkyne decomposition and carbon diffusion into the Pd lattice [42], appears to be a key factor in the selective hydrogenation of alkynes into alkenes. Thus, PdC_x_ species strongly disturbs the equilibrium between hydrogen dissolved in the bulk and adsorbed on the surface. The bulk hydrogen is much more reactive than the surface hydrogen, and can hydrogenate the adsorbed species upon their appearance on the surface, thus contributing to the unselective hydrogenation [44]. The incorporation of carbon into the upper Pd layers strongly influences the diffusion/transport of H from the bulk to the uppermost Pd layer, thereby hindering the participation of more energetic subsurface hydrogen in the catalytic process. On the other hand, increasing the hydrogen partial pressure promotes the unselective total hydrogenation of propyne to propane, which is accompanied by a decrease in the amount of surface PdC_x_ and an enhancement of the surface PdH_x_ species [45, 46]. Furthermore, the role of PdC_x_ was also deduced from *operando* EXAFS experiments [47]. In addition, these experiments proved the existence of different chemisorbed hydrogenated species depending on the dominant surface state (PdC_x_ or PdC_x_H_y_). That is, the chemisorbed hydrocarbon species are more dehydrogenated over PdC_x_ and more hydrogen rich over PdH_x_. Taking into account the above described processes, the general scheme of the hydrogenation of hydrocarbons with multiple unsaturations is linked to the so-called “rake mechanism” because the adsorption and desorption steps and equilibrium constant are like the “teeth” of a “comb” [48, 49]. This process can be accurately described taking into account the chemistry of the population of palladium surface sites and the rate constant (k) for each catalytic process [3]. Thus, two scenarios are considered: the partial hydrogenation on α-β PdC_x_ and the full hydrogenation on β-PdC_x_H_y_ catalysts, as shown in Fig. 5. These processes are governed by carbon and/or hydrogen subsurface atoms, as the *operando* experiments showed. Hence, the processes A and a are related to the adsorption and desorption of C_n_H_2n−2_ on the Pd surface yielding the formation of PdC_x_ as main product of the reaction. Moreover, in presence of C_n_H_n−2_ and H_2_, two reactions are expected, which are described by the constant processes B and C, given that in the case of low H_2_ concentration B is the dominant rate constant. Hence low partial pressures of hydrogen yields:

This reaction is selectively due to the formation of PdC_x_ by the incorporated carbon in the upper layers preventing the effective diffusion of rather reactive bulk/subsurface hydrogen. As a consequence, the upper carbonaceous species lead to the formation of alkenes avoiding the full hydrogenation on the Pd catalyst and thus hindering the C unselective mechanism. On the other hand, if the partial concentration of H_2_ is increased then C is the dominant prompting the hydrogenation of PdC_x_ as:

Once the PdC_x_ surface concentration is lowered and the PdH_x_ becomes dominant the reaction becomes unselective to the total hydrogenation to alkanes as follows:

Note that this reaction is not favored when PdC_x_ species are present because the carbonaceous species avoid the participation of subsurface hydrogen in the reaction. On the other hand, the unselective formation of alkanes (D, E) can be conducted when the concentration of chemisorbed hydrogen is high yielding the formation of surface PdH_x_. Therefore, β-PdH_x_ is formed facilitating the production of C_n_H_2n_ and C_n_H_2n+2_ due to the participation of more reactive subsurface hydrogen in the hydrogenation of alkynes.

## Conclusions

*On-line* gas analysis of propyne hydrogenation showed high propene selectivity at moderate conversion levels and propane formation near full conversion for a Pd-black model catalyst. This behavior is attributed to the population of hydrogen and carbon into the surface/subsurface regions that control the catalytic performance of Pd in the selective hydrogenation of propyne onto a PdH_x_ α-β phase. The selective/unselective hydrogenation is due to the fact that the incorporation of carbon into the Pd surface strongly influences the transport of hydrogen from the bulk to the surface and thus the surface species and events are decoupled from the bulk properties. PdC_x_ hinders the participation of more reactive subsurface hydrogen in the catalytic process hence making the selective semi-hydrogenation of alkynes to alkene possible. Otherwise, an increase in the partial pressure of H_2_ yields the progressive loss of PdC_x_ allowing the participation of subsurface hydrogen in the reaction and thus favoring the unselective hydrogenation to alkane onto a PdH_x_ β phase. Accordingly, these experiments at high pressure validate the previous reaction model demonstrated by in situ NAP-XPS at few mbar partial pressure and therefore close the “pressure gap” in the understanding of the hydrogenation of alkynes on Pd catalysts showing the evident difference between bulk and near-surface chemistry of the Pd catalyst in contact with both hydrogen and hydrocarbons. On the other hand, this work is an example for the value of true *operando* studies even and because it shows here the absence of a high-pressure gap due to a well understandable reason based in high sticking.

## Electronic supplementary material

Below is the link to the electronic supplementary material.

Supplementary material 1 (DOCX 564 KB)

## References

[1] Abelló S, Verboekend D, Bridier B, Pérez-Ramírez J (2008). Activated takovite catalysts for partial hydrogenation of ethyne, propyne, and propadiene. J Catal.

[2] McCue AJ, Anderson JA (2015). Recent advances in selective acetylene hydrogenation using palladium containing catalysts. Front Chem Sci Eng.

[3] Molnár Á, Sárkány A, Varga M (2001). Hydrogenation of carbon–carbon multiple bonds: chemo-, regio-and stereo-selectivity. J Mol Catal A.

[4] Chen B, Dingerdissen U, Krauter JGE, Rotgerink HL, Möbus K, Ostgard DJ, Panster P, Riermeier TH, Seebald S, Tacke H, Trauthwein H (2005). New developments in hydrogenation catalysis particularly in synthesis of fine and intermediate chemicals. Appl Catal A.

[5] Wehrli JT, Thomas DJ, Wainwright MS, Trimm DL, Cant NW (1991). Selective hydrogenation of propyne over supported copper catalysts: influence of support. Appl Catal.

[6] Stoltze P, Nørskov JK (1985). Bridging the” pressure gap” between ultrahigh-vacuum surface physics and high-pressure catalysis. Phys Rev Lett.

[7] Borodziński A, Bond GC (2008). Selective hydrogenation of ethyne in ethane-rich streams on palladium catalysts, Part 2: steady-state kinetics and effects of palladium particle size, carbon monoxide, and promoters. Catal Rev.

[8] Doyle AM, Shaikhutdinov SK, Jackson SD, Freund HJ (2003). Hydrogenation on metal surfaces: why are nanoparticles more active than single crystals?. Angew Chem Int Ed.

[9] Doyle AM, Shaikhutdinov SK, Freund HJ (2004). Alkene chemistry on the palladium surface: nanoparticles vs single crystals. J Catal.

[10] Wilde M, Fukutani K, Naschitzki M, Freund HJ (2008). Hydrogen absorption in oxide-supported palladium nanocrystals. Phys Rev B.

[11] Freund HJ (2005). Model studies on heterogeneous catalysts at the atomic level. Cat Today.

[12] Doyle AM, Shaikhutdinov SK, Freund HJ (2005). Surface-bonded precursor determines particle size effects for alkene hydrogenation on palladium. Angew Chem Int Ed.

[13] Silvestre-Albero J, Rupprechter G, Freund HJ (2005). Atmospheric pressure studies of selective 1, 3-butadiene hydrogenation on Pd single crystals: effect of CO addition. J Catal.

[14] Teschner D, Borsodi J, Wootsch A, Révay Z, Hävecker M, Knop-Gericke A, Jackson SD, Schlögl R (2008). The roles of subsurface carbon and hydrogen in palladium-catalyzed alkyne hydrogenation. Science.

[15] Teschner D, Révay Z, Borsodi J, Hävecker M, Knop-Gericke A, Schlögl R, Milroy D, Jackson SD, Torres D, Sautet P (2008). Understanding palladium hydrogenation catalysts: when the nature of the reactive molecule controls the nature of the catalyst active phase. Angew Chem Int Ed.

[16] Johnson AD, Daley SP, Utz AL, Ceyer ST (1992). The chemistry of bulk hydrogen-reaction of hydrogen embedded in nickel with adsorbed CH_3_. Science.

[17] Borodziński A, Bond GC (2006). Selective hydrogenation of ethyne in ethane-rich streams on palladium catalysts. Part 1. Effect of changes to the catalyst during reaction. Catal Rev.

[18] Wilde M, Fukutani K, Ludwig W, Brandt B, Fischer JH, Schauermann S, Freund HJ (2008). Influence of carbon deposition on the hydrogen distribution in Pd nanoparticles and their reactivity in olefin hydrogenation. Angew Chem Int Ed.

[19] Teschner D, Borsodi J, Kis Z, Szentmiklósi L, Révay Z, Knop-Gericke A, Schlögl R, Torres D, Sautet P (2010). Role of hydrogen species in palladium-catalyzed alkyne hydrogenation. J Phys Chem C.

[20] Ertl G (1991). Elementary steps in ammonia synthesis in catalytic ammonia synthesis.

[21] https://www.helmholtz-berlin.de/pubbin/igama_output?modus=einzel&sprache=en&gid=1607

[22] Velasco-Velez JJ, Pfeifer V, Hävecker M, Weatherup RS, Arrigo R, Chuang CH, Stotz E, Weinberg G, Salmeron M, Schlögl R, Knop-Gericke A (2015). Photoelectron spectroscopy at the graphene–liquid interface reveals the electronic structure of an electrodeposited cobalt/graphene electrocatalyst. Angew Chem Int Ed.

[23] Velasco-Vélez JJ, Pfeifer V, Hävecker M, Wang R, Centeno A, Zurutuza A, Algara-Siller G, Stotz E, Skorupska K, Teschner D, Kube P, Braeuninger-Weimer P, Hofmann S, Schlögl R, Knop-Gericke (2016). Atmospheric pressure X-ray photoelectron spectroscopy apparatus: bridging the pressure gap. Rev Sci Instrum.

[24] Suleiman M, Jisrawi NM, Dankert O, Reetz MT, Bähtz C, Kirchheim R, Pundt A (2003). Phase transition and lattice expansion during hydrogen loading of nanometer sized palladium clusters. J Alloys Compd.

[25] Vogel W, He W, Huang QH, Zou Z, Zhang XG, Yang H (2010). Palladium nanoparticles “breathe” hydrogen; a surgical view with X-ray diffraction. Int J Hydrog Energy.

[26] Chase ZA, Fulton JL, Camaioni DM, Mei D, Balasubramanian M, Pham VT, Zhao C, Weber RS, Wang Y, Lercher JA (2013). State of supported Pd during catalysis in water. J Phys Chem C.

[27] Fukai Y (1993). The metal-hydrogen system—basic bulk properties.

[28] Crespo-Quesada M, Yoon S, Jin M, Prestianni A, Cortese R, Cárdenas-Lizana F, Duca D, Weidenkaff A, Kiwi-Minsker L (2015). Shape-dependence of Pd nanocrystal carburization during acetylene hydrogenation. J Phys Chem C.

[29] Nag NK (2001). A study on the formation of palladium hydride in a carbon-supported palladium catalyst. J Phys Chem B.

[30] Phan TH, Schaak RE (2009). Polyol synthesis of palladium hydride: bulk powders vs. nanocrystals. Chem Commun.

[31] Lewis FA (1982). The palladium-hydrogen system. Platin Met Rev.

[32] Bugaev AL, Guda AA, Lomachenko KA, Shapovalov VV, Lazzarini A, Vitillo JG, Bugaev LA, Groppe E, Pellegrini R, Soldatov AV, Bokhoven JA, Lamberti C (2017). Core–shell structure of palladium hydride nanoparticles revealed by combined X-ray absorption spectroscopy and X-ray diffraction. J Phys Chem C.

[33] Narehood DG, Kishore S, Goto H, Adair JH, Nelson JA, Gutierrez HR, Eklund PC (2009). X-ray diffraction and H-storage in ultra-small palladium particles. Int J Hydrog Energy.

[34] Bugaev AL, Guda AA, Lazzarini A, Lomachenko KA, Groppo E, Pellegrini R, Piovano A, Emerich H, Soldanov AV, Dmitriev VP (2017). In situ formation of hydrides and carbides in palladium catalyst: when XANES is better than EXAFS and XRD. Catal Today.

[35] Noack K, Zbinden H, Schlögl R (1990). Identification of the state of palladium in various hydrogenation catalysts by XPS. Catal Lett.

[36] Teschner D, Vass E, Hävecker M, Zafeiratos S, Schnörch P, Sauer H, Knop-Gericke A, Schlögl R, Chaman M, Wootsch A, Canning AS, Gamman JJ, Jackson SD, McGregor J, Gladden LF (2006). Alkyne hydrogenation over Pd catalysts: a new paradigm. J Catal.

[37] Armbrüster M, Behrens M, Cinquini F, Föttinger K, Grin Y, Haghofer A, Penner S (2012). How to control the selectivity of palladium-based catalysts in hydrogenation reactions: the role of subsurface chemistry. ChemCatChem.

[38] Ai L, Zhang C, Chen Z (2011). Removal of methylene blue from aqueous solution by a solvothermal-synthesized graphene/magnetite composite. J Hazard Mater.

[39] Nikitin A, Ogasawara H, Mann D, Denecke R, Zhang Z, Dai H, Cho K, Nilsson A (2005). Hydrogenation of single-walled carbon nanotubes. Phys Rev Lett.

[40] Nikitin A, Li X, Zhang Z, Ogasawara H, Dai H, Nilsson A (2008). Hydrogen storage in carbon nanotubes through the formation of stable C–H bonds. Nano Lett.

[41] Brandt B, Fischer JH, Ludwig W, Libuda J, Zaera F, Schauermann S, Freund HJ (2008). Isomerization and hydrogenation of cis-2-butene on Pd model catalyst. J Phys Chem C.

[42] Shao L, Zhang B, Zhang W, Teschner D, Girgsdies F, Schlögl R, Su DS (2012). Improved selectivity by stabilizing and exposing active phases on supported Pd nanoparticles in acetylene-selective hydrogenation. Chem-A Eur J.

[43] Tew MW, Janousch M, Huthwelker T, van Bokhoven JA (2011). The roles of carbide and hydride in oxide-supported palladium nanoparticles for alkyne hydrogenation. J Catal.

[44] Neyman KM, Schauermann S (2010). Hydrogen diffusion into palladium nanoparticles: pivotal promotion by carbon. Angew Chem Int Ed.

[45] McCaulley JA (1993). In-situ X-ray absorption spectroscopy studies of hydride and carbide formation in supported palladium catalysts. J Phys Chem.

[46] García-Mota M, Bridier B, Pérez-Ramírez J, López N (2010). Interplay between carbon monoxide, hydrides, and carbides in selective alkyne hydrogenation on palladium. J Catal.

[47] Bauer M, Schoch R, Shao L, Zhang B, Knop-Gericke A, Willinger M, Schlögl R, Teschner D (2012). Structure–activity studies on highly active palladium hydrogenation catalysts by X-ray absorption spectroscopy. J Phys Chem C.

[48] Guo XC, Madix RJ (1995). Selective hydrogenation and HD exchange of unsaturated hydrocarbons on Pd (100)-p (1 × 1)-H (D). J Catal.

[49] Li D, Bui P, Zhao HY, Oyama ST, Dou T, Shen ZH (2012). Rake mechanism for the deoxygenation of ethanol over a supported Ni_2_P/SiO_2_ catalyst. J Catal.

