# Supplementary material for: The Role of Adsorbed and Subsurface Carbon Species for the Selective Alkyne Hydrogenation Over a Pd-Black Catalyst: An Operando Study of Bulk and Surface
Source: Top Catal. 2018 Oct 24;61(20):2052–61. doi: 10.1007/s11244-018-1071-6 (PMC6404787; doi:10.1007/s11244-018-1071-6)
Supplement: Supplementary file 1 — Supplementary material 1 (DOCX 564 KB) [file 11244_2018_1071_MOESM1_ESM.docx]

Supplementary information

**The role of adsorbed and subsurface carbon species for the selective alkyne hydrogenation over a Pd-black catalyst: An *operando* study of bulk and surface**

J. J. Velasco-Vélez^1,2,^*, D. Teschner^1,2^, F. Girgsdies^2^, M. Hävecker^1,2^, V. Streibel^2^, M. G. Willinger^2^, J. Cao^2^, M. Lamoth, E. Frei, R. Wang^3^, A. Centeno^4^, A. Zurutuza^4^, S. Hofmann^3^, R. Schlögl^1,2^ and A. Knop-Gericke^2^

***Corresponding author:** [velasco@fhi-berlin.mpg.de](mailto:velasco@fhi-berlin.mpg.de,)

^1^Department of Heterogeneous Reactions, Max Planck Institute for Chemical Energy Conversion, Mülheim an der Ruhr 45470, Germany

^2^Department of Inorganic Chemistry, Fritz-Haber-Institut der Max-Planck-Gesellschaft, Berlin 14195, Germany

^3^Department of Engineering, University of Cambridge, Cambridge CB3 0FA, United Kingdom

^4^Graphenea, San Sebastian 20018, Spain

*BET analysis*

The Pd balck powder was loaded in a glass rod (0.2903 g) and degassed 3 h at 80 °C and the surface area of the powder was estimated in a N_2_ atmosphere at 77K using a Brunauer–Emmett–Teller (BET) method using an Autosorb 6-MP from Quantachrome. The results are shown in figure S1 where the analysis correspond to a crystalline surface area of 38.255 m^2^/g.


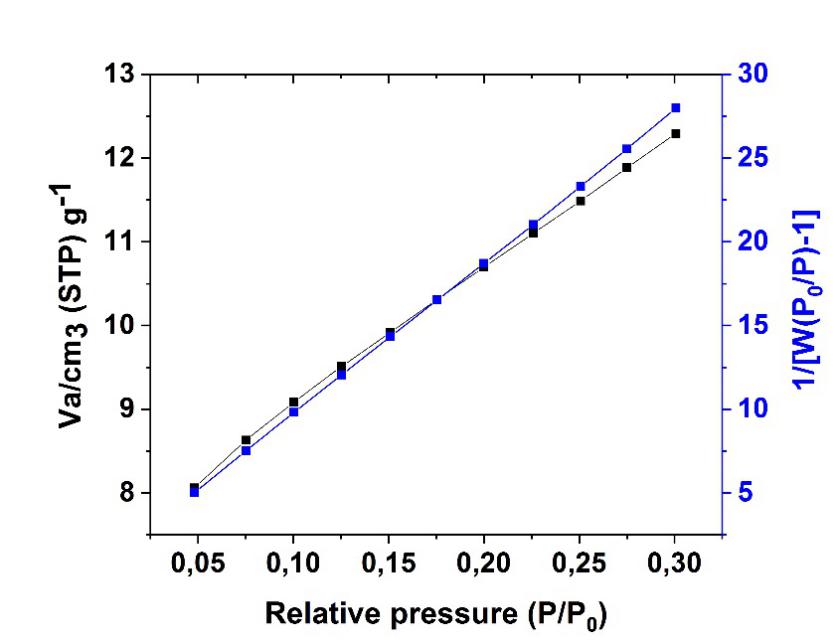


**Figure S1.** BET analysis of the Pd black samples.

*CO oxidation*

CO oxidation was performed in a self-constructed catalytic reactor setup equipped with an on-line gas-analyzer (X‑stream XE, Emerson/Rosemount, time resolution of 5 s) with an infrared sensor and a paramagnetic sensor, for the simultaneous quantification of oxygen, carbon monoxide, carbon dioxide and water. The CO gas was purified using a carbonyl remover consisting of a tube filled with inert silicon carbide heated up to 300 °C. The helium gas passed a water and oxygen filter patron (Air Liquide). The reactor was a quartz plug-flow U‑tube reactor with an inner diameter of 4 mm and 1 mm wall thickness. Loading of the reactor was performed with 10 mg catalyst diluted with 250 mg inert SiC surrounded by quartz wool on each side. CO oxidation was performed with 96 vol.‑% He, 2 vol.-% CO and 2 vol.-% O_2_ at 100 ml⋅min^-1^. The catalyst was pre-treated in synthetic air (21 % in He, 100 ml⋅min^‑1^) at 200 °C for 12 h. The catalytic oxidation was tested in a three cycle test with heating to 250 °C with 2 °C·min^-1^ and cooling to 30 °C between each cycle with a following 7 h dwell at 250 °C. Afterwards, the sample was kept at a constant temperature of 170 °C for 90 h. These measurements are shown in figure S2.


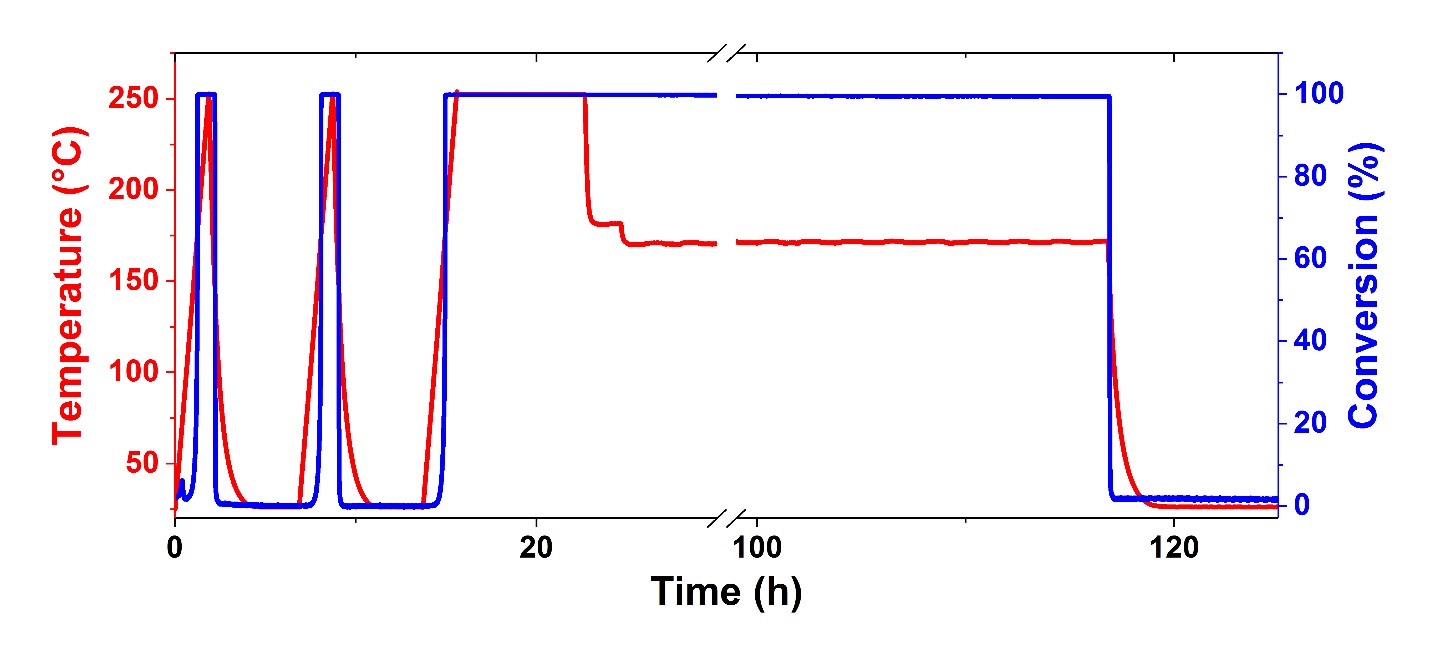


**Figure S2.** CO oxidation characterization depending on time and temperature.

Three heating-cooling processes from room-temperature up to 250 °C were accomplished. The first cycle was used to clean the catalyst surface and bulk. It was found that there is a 100 % conversion at 178 °C for the second and third cycles indicating an active and stable performance of the catalyst. After a 7 h dwell time at 250 °C subsequent to the third cycle, the samples was cooled down to 170 °C. Since the catalyst shows a 100 % conversion at 170 °C it can be concluded that the catalyst increased in activity with a drop for the temperature needed for 100 % conversion from 178 °C to 170 °C. During the 90 h dwell time the catalyst showed no deactivation indicating a high stability. Finally, the sample was cooled down to room-temperature yielding 100 % CO conversion until a temperature of 156 °C is reached, again indicating an activation behavior for the catalyst. These measurements indicate without a doubt that the catalyst is rather active and stable.

*In situ ESEM measurements*


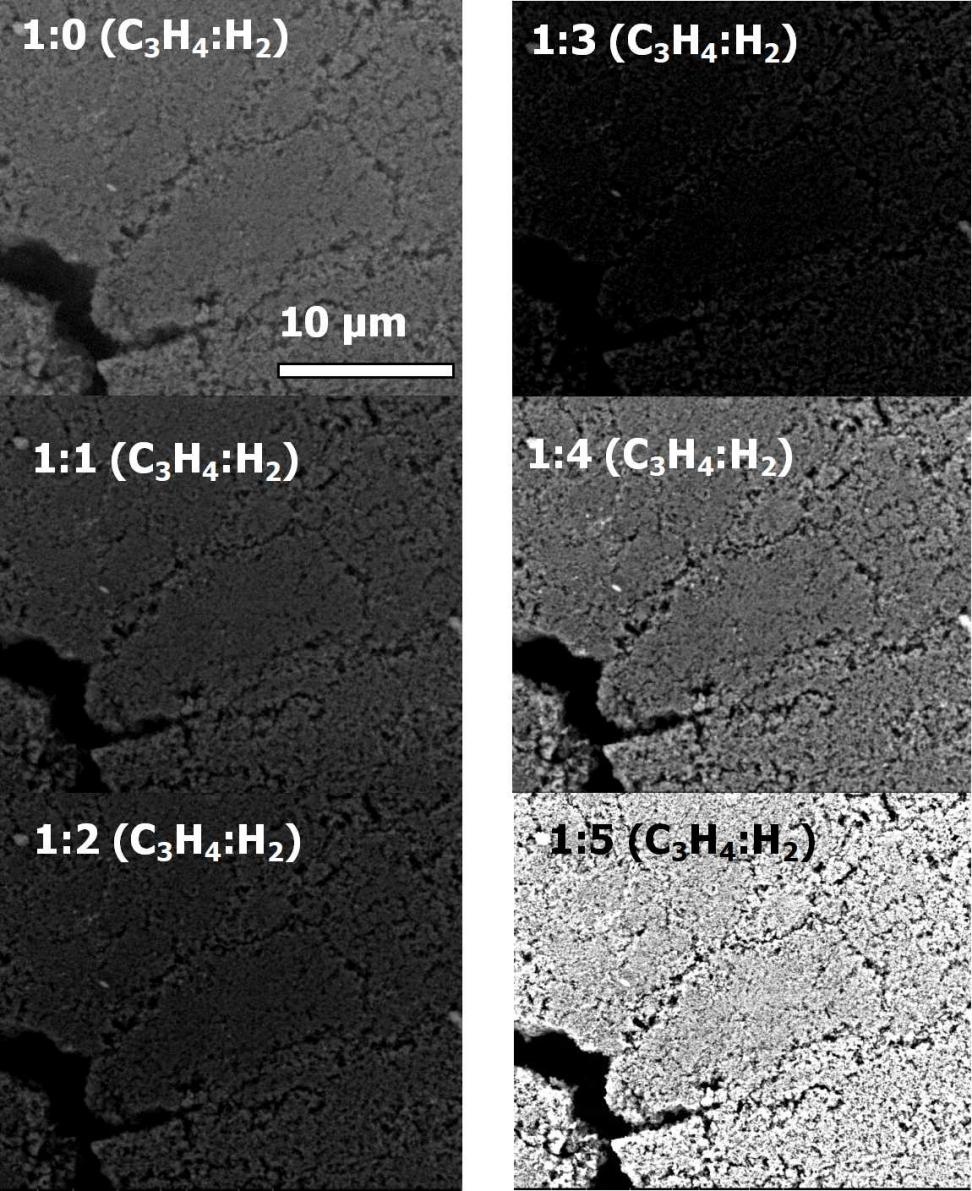


**Figure S4.** *In situ* ESEM measurements at 1 mbar partial pressure.

*In situ XRD measurements*


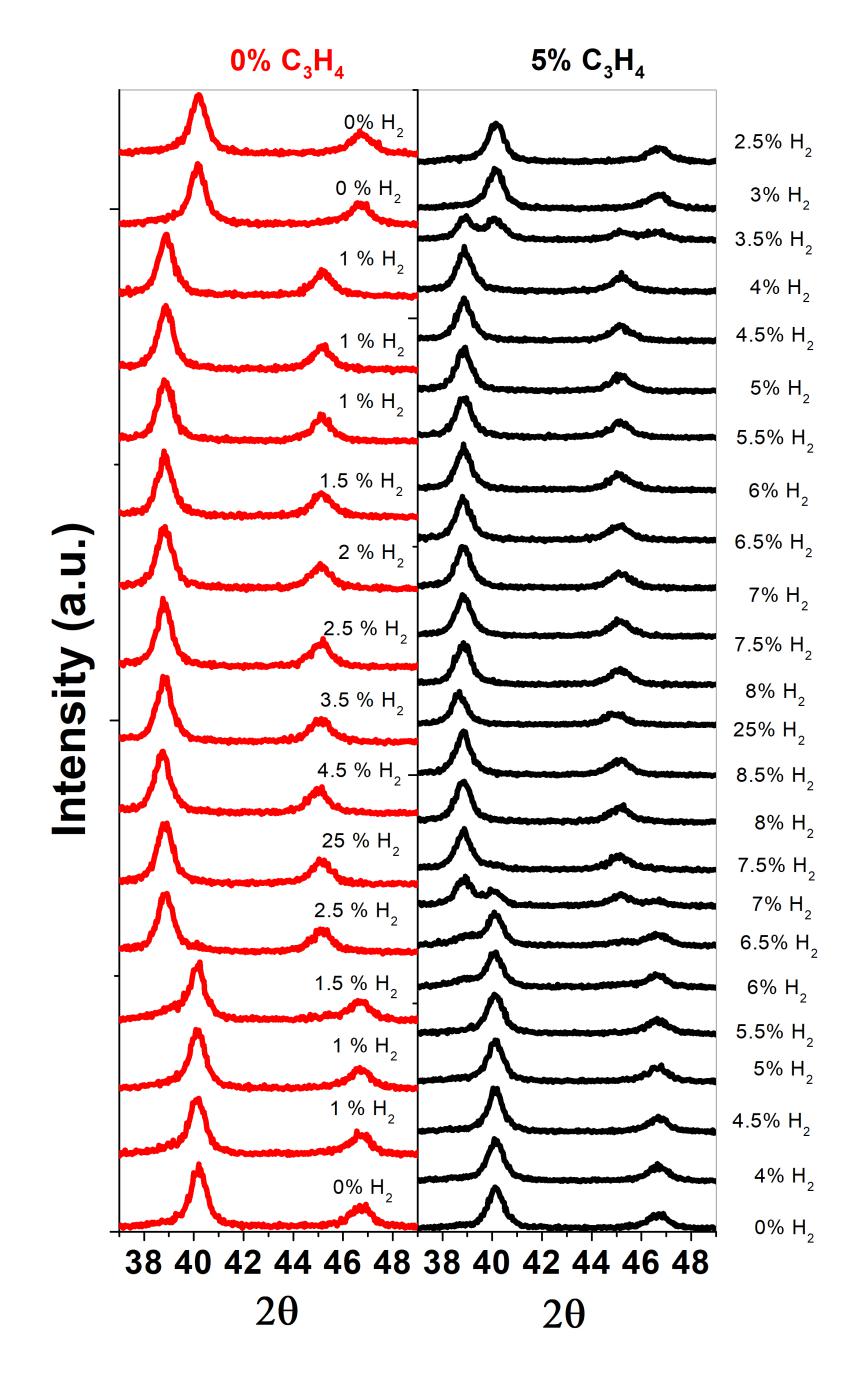


**Figure S5.** XRD measurements.
